# Effects of Omega-3 Polyunsaturated Fatty Acids on Brain Functions: A Systematic Review

**DOI:** 10.7759/cureus.30091

**Published:** 2022-10-09

**Authors:** Ibrahim M Dighriri, Abdalaziz M Alsubaie, Fatimah M Hakami, Dalal M Hamithi, Maryam M Alshekh, Fatimah A Khobrani, Fatimah E Dalak, Alanoud A Hakami, Efham H Alsueaadi, Laila S Alsaawi, Saad F Alshammari, Abdullah S Alqahtani, Ibrahim A Alawi, Amal A Aljuaid, Mohammed Q Tawhari

**Affiliations:** 1 Department of Pharmacy, King Abdulaziz Specialist Hospital, Taif, SAU; 2 Faculty of Medicine, Jazan University, Jazan, SAU; 3 Faculty of Nursing, Jazan University, Jazan, SAU; 4 Department of Pharmacy, Wadi Al Dawasir Security Forces Specialized Comprehensive Clinics, Wadi Al Dawasir, SAU; 5 Faculty of Pharmacy, Qassim University, Al Qassim, SAU; 6 Faculty of Pharmacy, Tabuk University, Tabuk, SAU; 7 Faculty of Pharmacy, King Khalid University, Abha, SAU; 8 Faculty of Pharmacy, Jazan University, Jazan, SAU; 9 Faculty of Pharmacy, Taif University, Taif, SAU; 10 Department of Pharmacy, Jazan General Hospital, Jazan, SAU

**Keywords:** fatty acids, epa, dha, pufas, mental health, brain, omega

## Abstract

Omega is a polyunsaturated fatty acid (PUFA) that has an essential impact on cognitive performance at all stages of life. Eicosapentaenoic acid (EPA), docosahexaenoic acid (DHA), and alpha-linolenic acid (ALA) are essential for brain functions. DHA, the dominant omega-3 in the brain, impacts neurotransmitters and functions of the brain. This systematic review aimed to assess the effects of omega-3 on brain functions. We searched for articles from 2010 to 2022 in PubMed, electronic databases: discover, academic search complete (EBSCO), and Cochrane. To increase search efficiency, search terms include database-specific indexed phrases and keywords. Search terms included "omega three," "DHA," "fish oil," "eicosapentaenoic acid," "EPA," "docosahexaenoic acid," "omega-3," "cognition," "brain," "mental health," and "PUFAs".We conducted a review of only randomized clinical trials (RCTs) that were published in English. We evaluated the quality of the studies using the Cochrane Collaboration bias assessment tool. Our search strategy yielded 174 articles, out of which 33 full-text articles were reviewed and nine articles were selected for data abstraction*.* The overall number of individuals in all nine studies was 1319. Of the participants, 591 (44.81%) were men, and 728 (55.19%) were women. Participants who received omega-3 were 700 (65.06%) compared to 376 (34.94%) who received a placebo, and their mean age was 45. Ingestion of omega-3 fatty acids increases learning, memory, cognitive well-being, and blood flow in the brain. Omega-3 treatments are advantageous, well-tolerated, and risk-free. Lonelier people, the elderly, and those who eat fewer healthy foods containing omega-3 may benefit from an omega-3 supplement. We suggest that natural omega-3 consumption through the diet should be promoted.

## Introduction and background

Omega-3 polyunsaturated fatty acids (PUFAs) are fatty acids whose chemical structure is double-bonded with three atoms in the opposite direction to the methyl group [1]. They are an essential component in lipid biotransformation in mammals. The three main types of omega-3, which are docosahexaenoic acid (DHA), alpha-linolenic acid (ALA), and eicosapentaenoic acid (EPA), are the most important in the human brain. DHA comprises approximately 40% of total fatty acids in the brain, while EPA comprises less than 1% of total brain acids [1,2].

Approximately 50-60% of the brain weight comprises lipids, of which 35% consists of omega-3 PUFAs. DHA accounts for more than 40% of total omega-3 PUFAs in neuronal tissue, especially in the gray matter [2,3]. In the later stages of pregnancy and the first 18 months of life, DHA accumulates rapidly in the brain [1,3]. DHA is often included in newborn formula due to its involvement in visual acuity and cognitive growth [4]. Endogenous DHA production in animals is restricted; therefore, breast milk provides an accessible supply for babies [3].

Omega-3 promotes cognition, neuronal preservation, and protection against neurodegeneration [5]. Neurotransmission is influenced by two mechanisms: changing membrane fluidity and increasing neurotransmitter release [6]. It also alleviates brain apoptosis through some mechanisms, such as decreasing responses to reactive oxygen species, which have an antiapoptotic action, or by up-regulating the expression of antiapoptotic proteins and down-regulating the expression of apoptotic proteins, leading to a slowing of the apoptosis reaction [7]. In this systematic review, our objective was to assess the effects of omega-3 on brain functions by reviewing the current literature. This review may also include a variety of contexts, people, and exposures, allowing for a more comprehensive knowledge of the effects of omega-3-PUFAs on brain functions.

## Review

Methodology

We followed systematic review guidelines in our study [8].

Research Question

Does omega-3 use affect brain function?

Sources of Data and Strategy for Search

Searches were carried out on PubMed, electronic databases: discover, academic search complete (EBSCO), and Cochrane from 2010 to 2022. To increase search efficiency, search terms include database-specific indexed phrases and keywords. Search terms included "omega three," "DHA," "fish oil," "eicosapentaenoic acid," "EPA," "docosahexaenoic acid," "omega-3," "cognition," "brain," "mental health," and "PUFAs." The search strategies were modified to suit each specific database. All the authors chose the studies. Duplicates were deleted, and inclusion criteria were applied; we examined the remaining papers at the title and abstract levels. Following that, the complete texts of the remaining records were obtained to determine whether they satisfied the inclusion criteria.

Inclusion and Exclusion Criteria

The inclusion criteria were randomized clinical trials (RCTs) that evaluated the effect of omega-3 on brain function. The population consisted of adult males and females, and the intervention was any omega-3 fatty acid treatment. The comparison was made between omega-3 and placebo. The outcomes were changes in brain functions. Non-randomized comparative studies, preclinical studies, reviews, observational studies, dissertations, quasi-experimental research, and studies in languages other than English were excluded. Only full publications and comprehensive studies were included.

Selection of Studies and Data Extraction

At the first screening, two authors independently read titles or abstracts. At the second screening, the full texts were read, and the data taken from the research was transferred to Excel software to the greatest extent practicable. We gathered the following information from each study: author and year of publication. The baseline parameters of the study included the number of participants, the dosage of omega-3 used, the major results of omega-3 effects on the brain, and the conclusion.

Quality Assessment of RCTs

The Cochrane collaboration's bias assessment instrument was employed to assess the quality of articles. RCTs were analyzed using the Cochrane bias checklist: low risk, unclear risk, or high risk of bias. No articles were excluded due to biased evaluations.

Ethical Considerations and Dissemination

This systematic study primarily used secondary data; therefore, as per our local IRB protocols, committee review and approval were deemed unnecessary and waived.

Result

**Figure 1:**
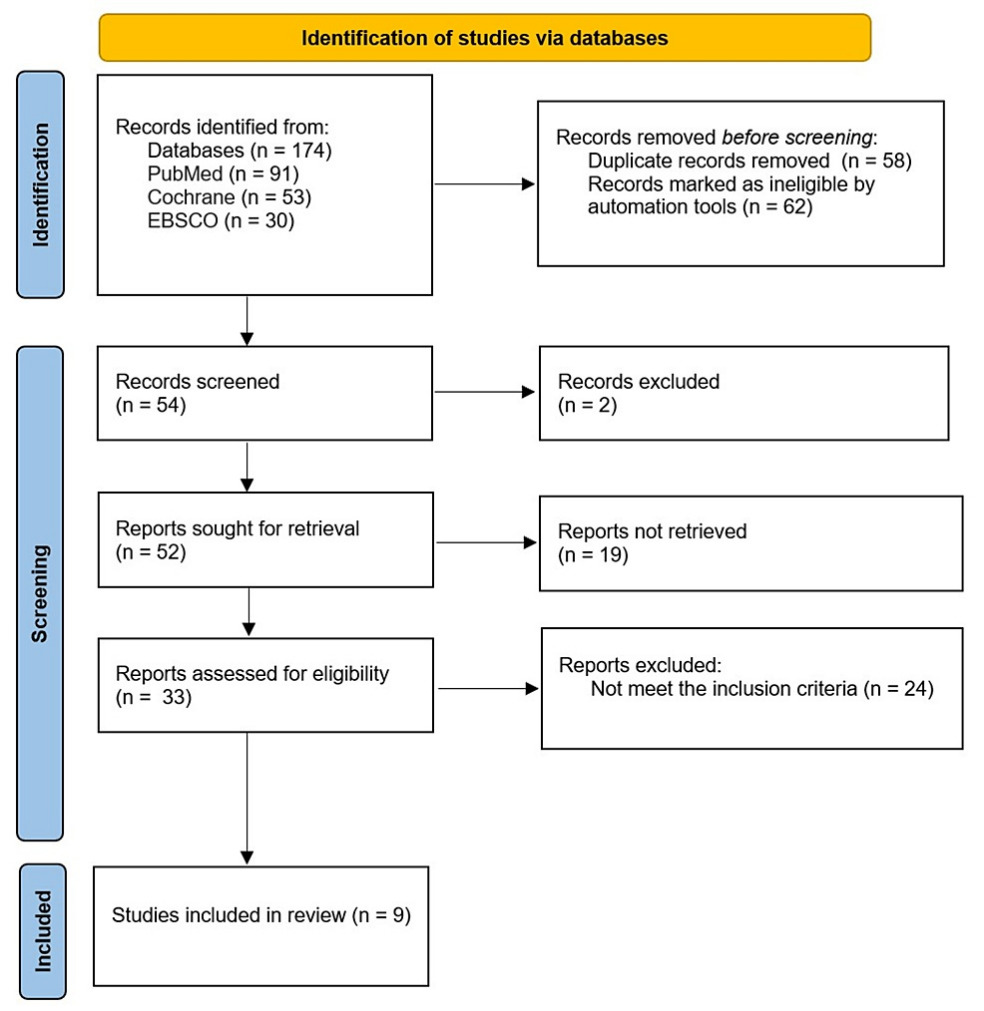
Diagram demonstrates the reporting elements recommended for systematic reviews. PRISMA flowchart: preferred reporting items for systematic reviews and meta-analyses flowchart, EBSCO: electronic databases: discover, academic search complete.

There were 174 articles in PubMed, Cochrane, and EBSCO publications that were detected for the effect of omega-3 on brain function. Fifty-eight duplicates and 62 ineligible articles were removed, and 54 articles were screened. Two unrelated abstracts and 19 reports not retrieved were eliminated. Thirty-three full-text papers were examined. Of these, 24 did not meet the inclusion requirements. The exclusion was due to cross-sectional, retrospective cohorts; articles in a non-English language; or the presence of some inputs that can cause bias, such as training, vitamins, and other nutrients. Nine articles met the study criterion for inclusion and contributed to our study (Figure 1).

**Figure 2:**
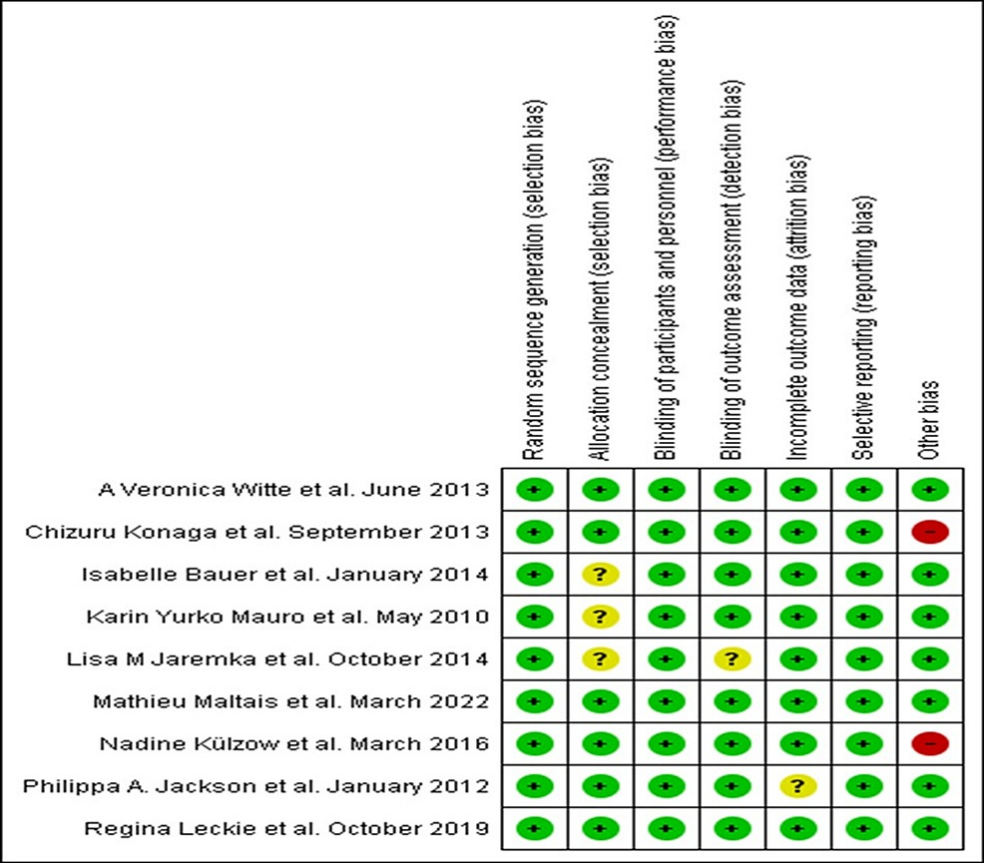
Bias assessment of each study. Green means little bias risk, yellow means uncertain bias risk, and red means high bias risk. Yurko-Mauro et al. [9]; Jackson et al. [10]; Witte et al. [11]; Konagai et al. [12]; Bauer et al. [13]; Jaremka et al. [14]; Külzow et al. [15]; Leckie et al. [16]; Maltais et al. [17].

Regarding the quality assessment of the articles, it revealed the following: all trials used appropriate randomization protocols; six experiments used the allocation concealment approach; all trials were double-blind; the outcome evaluation in eight RCTs was elaborated appropriately blinded; one trial had unclear bias due to insufficient outcome data; every RCT used-acceptable selective reporting; and in seven studies, the risk of other bias was low. The higher-quality trials were conducted by Witte et al., Maltais et al., and Leckie et al. (Figure 2).

The total number of individuals in all nine research papers that matched the inclusion criteria was 1319. Of the participants, 591 (44.81%) were men, and 728 (55.19%) were women. Participants who received omega-3 were 700 (65.06%) compared to 376 (34.94%) who received a placebo, and their mean age was 45.

At 24 weeks, there were substantially fewer six-type paired associate learning (PAL) errors connected to improvement in the DHA group than in the placebo group (difference rating, −1.63 ± 0.76 (−3.1, −0.14, 95% CI), P = 0.03). Consumption of DHA was rapid and late recognition memory grades (P<0.02) but not with better working memory or functioning evaluations. In participants taking DHA supplements, serum DHA concentrations quadrupled and were associated with better PAL scores (P<0.02). DHA was well-tolerated and recorded with no major side effects [9] (Table 1).

**Table 1:** The characteristics of the nine articles included in our study. PAL: paired associate learning; DHA: docosahexaenoic acid; EPA: eicosapentaenoic acid; RVIP: rapid visual information process; PUFAs: omega-3 polyunsaturated fatty acids; ANOVA: analysis of variance.

Author's name and publication date	Participant numbers and mean ages	Dose information	Main findings
Yurko-Mauro et al. [9], May 2010	Total number: 485 Mean age: 70	In this study, participants received 900 mg/day of DHA or a placebo.	At 24 weeks, there were substantially fewer PAL six-type errors with DHA than with placebo (difference rating, −1.63 ± 0.76 (−3.1, −0.14, 95% CI), P = 0.03). Consumption of DHA was connected to improved rapid and late recognition memory grades (P<0.02) but not with better work memory or functioning assessments. In participants with DHA, serum DHA concentrations quadrupled and were associated with better PAL scores (P<0.02). DHA was well-tolerated, and no major side effects were recorded. A 24-week period of a 900 mg/day dose of DHA enhanced learning and memory performance in patients and is a valuable supplement that improves cognitive health as people age.
Jackson et al. [10], January 2012	Total number: 65 (16 men and 49 women) Age range: 1-29 Mean age: 20	This study compared the impact of 12 weeks of daily docosahexaenoic acid in fish oil (1 g or 2 g) versus placebo supplements (olive oil).	During cognitive activities, supplementation with both doses of fish oil resulted in considerably higher levels of oxyhemoglobin and overall hemoglobin levels, indicating improved blood circulation. Changes did not consistently follow variations in hemodynamic response to activities in cognitive function. The ANOVA revealed that fish oil treatment had a significant impact on oxyhemoglobin (F(2, 59) = 3.54, P<0.05). Post-hoc analyses of total treatment indicated that the 2 g fish oil group had substantially higher levels of oxyhemoglobin when doing an activity (P<0.05) than the placebo group. According to the comparisons performed, 2 g of fish oil was significantly (all P<0.001) performance-related and elevated total hemoglobin during each activity. Still, substantially higher levels of total hemoglobin were only observed during the Stroop and RVIP task periods for the 1 g group of fish oil. In contrast to the placebo group, consuming both doses of fish oil resulted in a considerable rise in oxyhemoglobin and total hemoglobin levels, indicating greater blood circulation during cognitive activities. Effects on cognitive function did not consistently follow hemodynamic changes in activity.
Witte et al. [11], June 2013	Total number: 65 Age range: 50-75 Mean age: 63.9	In this study, participants received 2.2 g/day of fish oil for 26 weeks (four daily pills), and the placebo capsules were sunflower.	Subjects in the omega-3 group had a significantly higher percentage of DHA and EPA assessed on erythrocyte membranes in blood plasma after treatment, compared to controls. Supplementation caused a rise in EPA percentages (Wilcoxon test, T = 4.3, P<0.001), while a reduction was indicated in the placebo group (Wilcoxon test, T = −3.2, P<0.05). Post-hoc t-tests revealed that the omega-3 supplement improved executive functions by 26%, while the placebo did not affect performance. Regarding composite memory grade, both groups had a comparable impact on the retest at follow-up with no significant group impact. This interventional investigation found that after 26 weeks of excessive amounts of marine omega-3, the executive performance of older adults improved compared to the placebo group. Overall, omega-3 and fasting insulin levels were linked to cognitive improvements.
Konagai et al. [12], September 2013	Total number: 45 (all male) Age range: 61-72	This study used three different kinds of experimental additives: krill oil as a PUFAS-rich oil mixed with phosphatidylcholine, sardine oil, and triglycerides as a placebo.	For 12 weeks, the groups that were given krill oil or sardine oil exhibited higher oxyhemoglobin content in channel ten than in the group that was given triglycerides. The krill oil group showed considerably higher oxyhemoglobin levels in the brain's frontal region than the triglyceride sample. This research suggests that n-3 PUFAs improve cognition in the elderly.
Bauer et al. [13], January 2014	Total number: 13 (four men and nine women) Age range: 20-24	This supplementation study used two distinct fish oil meals. The first diet (Eye-Q^TM^, Novasel) contained a high EPA: DHA ratio, while the second diet (Efalex^TM^, Efamol) had a high DHA: EPA ratio. Participants took six pills daily as a supplement.	The results showed a decrease in the functional activity of the cingulate cortex and an improvement in the right precentral gyrus after the EPA-rich dose, as well as a reduction in response times on the Stroop task with color words Stroop. On the contrary, DHA increased right precentral gyrus activity in spatial cognition tasks but did not affect behavioral performance. It was found that after EPA-rich supplementation, individuals' brains performed with less difficulty and had higher cognitive performance than before supplementation. In contrast, the increase in functional induction and the absence of progress in cognitive performance in time after DHA may demonstrate that DHA is much less helpful than EPA in improving neurocognitive functioning after one month.
Jaremka et al. [14], October 2014	Total number: 138 (67% female) Age range: 51.04	In this study, participants received 1.25 g of omega-3 or 2.5 g of omega-3 per day for four months.	Lonelier participants in the placebo group exhibited worse episodic memory after dosage, as judged by instant (b = 0.28, t (117) = 2.62, p = 0.010) and long delay (b = 0.06, t (116) = 2.07, p = 0.040) free recall. This impact was not observed in the 1.25 g/day or 2.5 g/day supplementation groups, with p-values greater than 10. Plasma omega-ratio data confirmed these findings. There was no difference between omega-3 on short-delay recollection or other cognitive tests associated with loneliness, with all p-values greater than 0.32. The loneliness impact was not found in people taking 1.25 g or 2.5 g of omega-3 daily. Compared to the placebo group, lonelier individuals demonstrated greater instant free recall when taking the omega-3 supplement dose of 2.5 g/day. Increases in the plasma omega-6: the omega-3 ratio was associated with moderately superior immediate and long-latency-free recall in lonelier subjects. Lonelier people lose episodic memory over time. Loneliness-related episodic memory impairments were reduced by omega-3 supplementation, especially at a high dose (2.5 g/day). Lonelier people who received the placebo showed lower episodic verbal memory than less lonely ones, regardless of baseline scores. Users of omega-3 did not have this impact. Lonelier supplement users exhibited stronger verbal episodic memory than placebo users. Supplements boosted verbal and episodic memory.
Külzow et al. [15], March 2016	Total number: 44 (all female) Age range: 50-75	In this study, the omega-3 fatty acid group was given four fish-oil pills daily for 26 weeks. Four pills daily included 2200 mg of omega-3. The placebo group received four daily pills containing 1015 mg of sunflower oil.	An increase in omega-3 indexes, higher proportions of EPA, and DHA in peripheral blood erythrocytes led to a significantly enhanced recall of relevant object location associations for the omega-3 group versus the placebo group, without significantly changing learning and response time. Omega-3 improves the memory of elderly people. Omega interventions are valuable, well-tolerated, and safe.
Leckie et al. [16], October 2019	Total number: 271 (118 men and 153 women) Age range: 30-54 Mean age: 43	Participants in this study received 300 mg per day of omega-3, which was taken for 18 weeks via fish-oil capsules, or they received a placebo.	EPA and DHA concentrations in red blood cells rose as predicted, and capsule compliance was greater than 95%. No cognitive domain was affected by the supplement. Fish-oil treatment improved executive function in people with low baseline DHA levels compared with the placebo group. No changes in brain morphology were observed. A moderate dose of omega-3 for a moderate period did not impact neuropsychological function or brain morphology.
Maltais et al. [17], March 2022	Total number: 193 Age range: 20-80	Supplemental omega-3 is taken via four pills per day. Placebo pills contain high-oleic soybean or corn oil with no DHA or EPA.	After six months of omega-3, four cognitive domains of healthy participants did not vary between groups. Participants with low episodic memory improved significantly with omega-3 (p = 0.043). Cognitively healthy people did not benefit from a six-month dose of omega-3, and apolipoprotein E status or age had no influence.

The analysis of variance (ANOVA) revealed that fish oil treatment had a significant impact (F (2, 59) = 3.54, P<0.05). Post-hoc analyses of the total treatment mean indicated that the 2 g fish oil group had substantially higher oxyhemoglobin levels during task performance (P<0.05) than the placebo group. According to the comparisons made, the 2 g of fish oil group had significantly (all P<0.001) higher levels of total hemoglobin during the performance of each activity. However, significantly higher levels of total hemoglobin were only observed during the Stroop and rapid visual information processing task (RVIP) periods for the 1 g fish oil group [10].

Subjects in the omega-3 group had a significantly higher percentage of DHA and EPA, assessed on erythrocyte membranes in blood plasma after treatment, compared to controls. Supplementation caused a rise in EPA percentages (Wilcoxon test, T = 4.3, P<0.001), while a reduction was indicated in the placebo group (Wilcoxon test, T = −3.2, P<0.002). Modifications in DHA levels were not statistically significant (P>0.05). Post-hoc t-tests revealed that the omega-3 supplement improved executive functions by 26%, while the placebo did not affect performance. Regarding composite memory grade, both groups had a comparable impact on the retest at follow-up with no significant group impact [11].

For 12 weeks, the groups that took krill oil or sardine oil exhibited more significant increases in the oxyhemoglobin content in channel ten than the group that took medium-chain triglycerides. Compared to the medium-chain triglyceride sample, the krill oil group demonstrated considerably more significant changes in oxyhemoglobin levels in the left frontal region. It has been previously documented that n-3 PUFAs increase geriatric cognitive function [12].

Both supplements reduced the proportion of arachidonic acid to EPA levels. After the EPA-rich dose, a functional decline in the anterior cingulate cortex led to increased activity in the right precentral gyrus. It decreased response times for the Stroop color-word task. On the contrary, DHA supplementation increased functional activity in the right precentral gyrus and spatial cognition tasks but did not affect behavioral performance [13].

Lonelier people in the placebo group exhibited worse episodic memory after dosage, as judged by instant (b = 0.28, t (117) = 2.62, p = 0.010) and long-delayed (b = 0.06, t (116) = 2.07, p = 0.040) free recall. This impact was not seen in the 1.25-g/day or 2.5-g/day supplementation groups, with all p values greater than 10. Plasma omega-ratio data confirmed these findings. Omega-3 did not affect short-delay recollection or other cognitive examinations associated with loneliness, with p-values greater than 0.32 [14].

An increase in omega-3 indexes, higher proportions of EPA, and DHA in peripheral blood erythrocytes led to a significantly enhanced recall of relevant object location associations for omega-3 versus placebo without significantly changing learning and response time. These findings suggest that omega-3 improves memory in the elderly [15].

EPA and DHA concentrations in red blood cells rose as predicted, and capsule compliance was greater than 95%. No cognitive domain was affected by the supplement. Fish oil treatment improved executive function in people with low baseline DHA levels compared to a placebo group. No changes in brain morphology were observed [16]. After six months of omega-3, four cognitive domains of healthy participants did not vary between groups. Participants with low episodic memory scores improved significantly with omega-3 (p = 0.043) [17] (Table 1).

Discussion

Our results revealed that supplementing with omega-3 fatty acids seems to alter cognitive processes in humans. Intense supplementation with omega-3 fatty acids has been shown to improve mental performance. The use of omega-3 leads to higher hemoglobin oxygen saturation and total hemoglobin concentrations, suggesting an improvement in blood circulation in the brain. The findings of our study are in accordance with previous experiments, such as previous research suggesting that DHA intake may help improve early memory and learning deficiencies related to cognitive aging [18]. Furthermore, several studies revealed potential consequences of omega-3 consumption on mental performance in different age groups, particularly young participants [13,19] or older participants [15,20]. Omega-3 PUFAs, often present in oily fish, are necessary for proper brain growth and maintenance [2]. Omega-3 fatty acids help to inhibit neuronal cell death [21,22]. Moreover, they lower inflammation [23] and affect brain functions [24].

Our results demonstrate that with EPA supplementation, individuals' brains display higher cognitive performance. On the contrary, the increase in functional induction and the absence of progress in cognitive performance after DHA may demonstrate that DHA is much less helpful than EPA in improving neurocognitive functioning. According to the findings, 900 mg of DHA can be used as a dietary neuroprotective agent to treat specific early cognitive impairments. Such mental abnormalities are likely to develop due to natural aging or be observed before diagnosing mild cognitive impairment [9]. From 1992 to 2006, a prospective trial of 1880 participants found that a Mediterranean diet rich in fish, vegetables, and fruits, along with regular physical exercise, reduced the incidence of cognitive impairment [25]. Preclinical and clinical investigations have shown that increased DHA-rich fish consumption reduces the onset of dementia and mental deterioration [26-28].

Our findings showed that a moderate dose of omega-3 taken for a moderate period had no impact on neuropsychological function or brain morphology. Significant alterations in these other cognitive areas are conceivable with much more severe cognitive decline or prolonged omega-3 administration. DHA is required for neuronal growth and a variety of brain activities [3]. On the contrary, a 2008 RCT found that 26 weeks of EPA and DHA intake had no overall impact on cognitive function [29]. Past studies suggest that smaller omega-3 doses may have led to restricted omega-3 brain transport and that the presence of apolipoprotein E4 (APOE4) also influences the response to DHA intake [30]. DHA and EPA levels in cerebrospinal fluid reduction in APOE4 carriers after DHA administration might be due to decreased brain absorption or increased brain utilization [30,31]. Dose and duration are essential variables that define DHA transport to tissues. One study found that plasma phospholipids were at saturation after six weeks of 2 g/day DHA administration [32]. Furthermore, adherence to omega-3 and metabolism might vary between individuals [33]. Additionally, previous research showed that serum omega-3 response varied with age, with higher amounts in individuals aged <60 than those aged <40. This indicates that omega-3 will improve cognitive function in the elderly [17].

In this investigation, lonelier participants showed reduced episodic memory over time. Loneliness-induced episodic memory impairments were reduced by omega-3 supplementation, especially at a high dosage. According to longitudinal aging research, lonelier people aged >65 exhibited more significant decreases in episodic verbal memory for four years than those who were less lonely [34]. However, research showed that omega-3 improved immediate verbal memory in adults with minor cognitive impairments, indicating that omega-3 may have the ability to help lonely individuals struggling with a more significant mental deterioration over time [13,14].

Omega-3 interventions are valuable, well-tolerated, and safe. According to a past study, DHA is well-tolerated and has a considerable favorable impact on progressive memory loss, a serious health problem among the elderly [9]. Omega-3 supplementation increased phosphatide levels in the brain, increased neurotransmitter release, and improved cognition [35]. Studies on omega-3 dietary shortages demonstrated how a decrease in brain DHA might cause changes in neuronal membrane characteristics [36], change enzyme activity and electrophysiological qualities [37], change neurotransmission [38], and reduce memory performance [38].

Some suggest cardiovascular disease, especially hypertension, is a risk factor for cognitive problems such as Alzheimer’s [39]. Previous research found that DHA consumption caused a substantial drop-in heart rate, which might help lower the risk of catastrophic cardiac events [40]. The Food and Drug Administration (FDA) has ruled that omega-3 formulations have been shown to reduce cardiovascular risk in selected individuals with mild hypertriglyceridemia safely. The FDA contends that food labeling should state that consuming omega-3 in food or dietary products may lower the risk of high blood pressure and coronary disease. They also advise that adults ingest no more than 3 g of omega-3 per day, with supplements delivering no more than 2 g [41].

This work had two main limitations: (1) Much of the research did not look at the potential confounding or mediating impacts of other crucial components interacting with the metabolic system, such as other nutrients and vitamins. (2) Our review included only nine articles that were found by searching just three databases.

## Conclusions

Consumption of omega-3 improved learning, memory ability, cognitive well-being, and blood flow in the brain. Omega-3 therapies are beneficial, well-tolerated, and very low-risk. Lonelier people, the elderly, and people with less consumption of healthy foods containing omega-3 can benefit from the consumption of omega-3 supplements. We suggest that the natural intake of omega-3 through food should be encouraged. Fish have the highest concentrations of DHA and EPA. The FDA recommends consuming 3 g of omega-3 daily, with dietary supplements that deliver up to 2 g per day.

## References

[1] Gharami K, Das M, Das S (2015). Essential role of docosahexaenoic acid towards development of a smarter brain. Neurochem Int.

[2] Bos DJ, van Montfort SJ, Oranje B, Durston S, Smeets PA (2016). Effects of omega-3 polyunsaturated fatty acids on human brain morphology and function: what is the evidence?. Eur Neuropsychopharmacol.

[3] Fang X, Sun W, Jeon J (2020). Perinatal docosahexaenoic acid supplementation improves cognition and alters brain functional organization in piglets. Nutrients.

[4] Brew BK, Toelle BG, Webb KL, Almqvist C, Marks GB (2015). Omega-3 supplementation during the first 5 years of life and later academic performance: a randomised controlled trial. Eur J Clin Nutr.

[5] Wang L, Fan H, He J, Wang L, Tian Z, Wang C (2018). Protective effects of omega-3 fatty acids against Alzheimer's disease in rat brain endothelial cells. Brain Behav.

[6] Heras-Sandoval D, Pedraza-Chaverri J, Pérez-Rojas JM (2016). Role of docosahexaenoic acid in the modulation of glial cells in Alzheimer's disease. J Neuroinflammation.

[7] Mayurasakorn K, Williams JJ, Ten VS, Deckelbaum RJ (2011). Docosahexaenoic acid: brain accretion and roles in neuroprotection after brain hypoxia and ischemia. Curr Opin Clin Nutr Metab Care.

[8] Moher D, Liberati A, Tetzlaff J, Altman DG (2009). Preferred reporting items for systematic reviews and meta-analyses: the PRISMA statement. J Clin Epidemiol.

[9] Yurko-Mauro K, McCarthy D, Rom D (2010). Beneficial effects of docosahexaenoic acid on cognition in age-related cognitive decline. Alzheimers Dement.

[10] Jackson PA, Reay JL, Scholey AB, Kennedy DO (2012). Docosahexaenoic acid-rich fish oil modulates the cerebral hemodynamic response to cognitive tasks in healthy young adults. Biol Psychol.

[11] Witte AV, Kerti L, Hermannstädter HM (2014). Long-chain omega-3 fatty acids improve brain function and structure in older adults. Cereb Cortex.

[12] Konagai C, Yanagimoto K, Hayamizu K, Han L, Tsuji T, Koga Y (2013). Effects of krill oil containing n-3 polyunsaturated fatty acids in phospholipid form on human brain function: a randomized controlled trial in healthy elderly volunteers. Clin Interv Aging.

[13] Bauer I, Hughes M, Rowsell R, Cockerell R, Pipingas A, Crewther S, Crewther D (2014). Omega-3 supplementation improves cognition and modifies brain activation in young adults. Hum Psychopharmacol.

[14] Jaremka LM, Derry HM, Bornstein R (2014). Omega-3 supplementation and loneliness-related memory problems: secondary analyses of a randomized controlled trial. Psychosom Med.

[15] Külzow N, Witte AV, Kerti L, Grittner U, Schuchardt JP, Hahn A, Flöel A (2016). Impact of omega-3 fatty acid supplementation on memory functions in healthy older adults. J Alzheimers Dis.

[16] Leckie RL, Lehman DE, Gianaros PJ (2020). The effects of omega-3 fatty acids on neuropsychological functioning and brain morphology in mid-life adults: a randomized clinical trial. Psychol Med.

[17] Maltais M, Lorrain D, Léveillé P (2022). Long-chain omega-3 fatty acids supplementation and cognitive performance throughout adulthood: a 6-month randomized controlled trial. Prostaglandins Leukot Essent Fatty Acids.

[18] Blackwell AD, Sahakian BJ, Vesey R, Semple JM, Robbins TW, Hodges JR (2004). Detecting dementia: novel neuropsychological markers of preclinical Alzheimer's disease. Dement Geriatr Cogn Disord.

[19] Danthiir V, Hosking DE, Nettelbeck T (2018). An 18-mo randomized, double-blind, placebo-controlled trial of DHA-rich fish oil to prevent age-related cognitive decline in cognitively normal older adults. Am J Clin Nutr.

[20] Baleztena J, Ruiz-Canela M, Sayon-Orea C (2018). Association between cognitive function and supplementation with omega-3 PUFAs and other nutrients in ≥ 75 years old patients: a randomized multicenter study. PLoS One.

[21] Kidd PM (2007). Omega-3 DHA and EPA for cognition, behavior, and mood: clinical findings and structural-functional synergies with cell membrane phospholipids. Altern Med Rev.

[22] Wärnberg J, Gomez-Martinez S, Romeo J, Díaz LE, Marcos A (2009). Nutrition, inflammation, and cognitive function. Ann N Y Acad Sci.

[23] Kiecolt-Glaser JK, Belury MA, Andridge R, Malarkey WB, Hwang BS, Glaser R (2012). Omega-3 supplementation lowers inflammation in healthy middle-aged and older adults: a randomized controlled trial. Brain Behav Immun.

[24] Rafnsson SB, Deary IJ, Smith FB, Whiteman MC, Rumley A, Lowe GD, Fowkes FG (2007). Cognitive decline and markers of inflammation and hemostasis: the Edinburgh Artery Study. J Am Geriatr Soc.

[25] Scarmeas N, Luchsinger JA, Schupf N, Brickman AM, Cosentino S, Tang MX, Stern Y (2009). Physical activity, diet, and risk of Alzheimer disease. JAMA.

[26] Florent-Béchard S, Desbène C, Garcia P (2009). The essential role of lipids in Alzheimer's disease. Biochimie.

[27] Yurko-Mauro K (2010). Cognitive and cardiovascular benefits of docosahexaenoic acid in aging and cognitive decline. Curr Alzheimer Res.

[28] Calon F, Lim GP, Yang F (2004). Docosahexaenoic acid protects from dendritic pathology in an Alzheimer's disease mouse model. Neuron.

[29] van de Rest O, Geleijnse JM, Kok FJ (2008). Effect of fish oil on cognitive performance in older subjects: a randomized, controlled trial. Neurology.

[30] Arellanes IC, Choe N, Solomon V (2020). Brain delivery of supplemental docosahexaenoic acid (DHA): a randomized placebo-controlled clinical trial. EBioMedicine.

[31] Bazinet RP, Metherel AH, Chen CT, Shaikh SR, Nadjar A, Joffre C, Layé S (2020). Brain eicosapentaenoic acid metabolism as a lead for novel therapeutics in major depression. Brain Behav Immun.

[32] Arterburn LM, Hall EB, Oken H (2006). Distribution, interconversion, and dose response of n-3 fatty acids in humans. Am J Clin Nutr.

[33] Kiecolt-Glaser JK, Epel ES, Belury MA (2013). Omega-3 fatty acids, oxidative stress, and leukocyte telomere length: a randomized controlled trial. Brain Behav Immun.

[34] Shankar A, Hamer M, McMunn A, Steptoe A (2013). Social isolation and loneliness: relationships with cognitive function during 4 years of follow-up in the English Longitudinal Study of Ageing. Psychosom Med.

[35] Wurtman RJ, Cansev M, Sakamoto T, Ulus IH (2009). Use of phosphatide precursors to promote synaptogenesis. Annu Rev Nutr.

[36] Eldho NV, Feller SE, Tristram-Nagle S, Polozov IV, Gawrisch K (2003). Polyunsaturated docosahexaenoic vs docosapentaenoic acid-differences in lipid matrix properties from the loss of one double bond. J Am Chem Soc.

[37] Bourre JM, Francois M, Youyou A, Dumont O, Piciotti M, Pascal G, Durand G (1989). The effects of dietary alpha-linolenic acid on the composition of nerve membranes, enzymatic activity, amplitude of electrophysiological parameters, resistance to poisons and performance of learning tasks in rats. J Nutr.

[38] Lim SY, Hoshiba J, Salem N Jr (2005). An extraordinary degree of structural specificity is required in neural phospholipids for optimal brain function: n-6 docosapentaenoic acid substitution for docosahexaenoic acid leads to a loss in spatial task performance. J Neurochem.

[39] Stampfer MJ (2006). Cardiovascular disease and Alzheimer's disease: common links. J Intern Med.

[40] Mori TA, Bao DQ, Burke V, Puddey IB, Beilin LJ (1999). Docosahexaenoic acid but not eicosapentaenoic acid lowers ambulatory blood pressure and heart rate in humans. Hypertension.

[41] (2022). FDA announces new qualified health claims for EPA and DHA omega-3 consumption and the risk of hypertension and coronary heart disease | FDA. https://www.fda.gov/food/cfsan-constituent-updates/fda-announces-new-qualified-health-claims-epa-and-dha-omega-3-consumption-and-risk-hypertension-and..

